# Depletion of tRNA-halves enables effective small RNA sequencing of low-input murine serum samples

**DOI:** 10.1038/srep37876

**Published:** 2016-11-30

**Authors:** Alan Van Goethem, Nurten Yigit, Celine Everaert, Myrthala Moreno-Smith, Liselot M. Mus, Eveline Barbieri, Frank Speleman, Pieter Mestdagh, Jason Shohet, Tom Van Maerken, Jo Vandesompele

**Affiliations:** 1 Center for Medical Genetics Ghent (CMGG), Ghent University, Ghent, Belgium; 2 Cancer Research Institute Ghent (CRIG), Ghent University Ghent, Belgium; 3 Bioinformatics Institute Ghent (BIG), Ghent University, Ghent, Belgium; 4 Department of Pediatrics, Section of Hematology-Oncology, Texas Children’s Cancer Center, Baylor College of Medicine, Houston, Texas, United States

## Abstract

The ongoing ascent of sequencing technologies has enabled researchers to gain unprecedented insights into the RNA content of biological samples. MiRNAs, a class of small non-coding RNAs, play a pivotal role in regulating gene expression. The discovery that miRNAs are stably present in circulation has spiked interest in their potential use as minimally-invasive biomarkers. However, sequencing of blood-derived samples (serum, plasma) is challenging due to the often low RNA concentration, poor RNA quality and the presence of highly abundant RNAs that dominate sequencing libraries. In murine serum for example, the high abundance of tRNA-derived small RNAs called 5′ tRNA halves hampers the detection of other small RNAs, like miRNAs. We therefore evaluated two complementary approaches for targeted depletion of 5′ tRNA halves in murine serum samples. Using a protocol based on biotinylated DNA probes and streptavidin coated magnetic beads we were able to selectively deplete 95% of the targeted 5′ tRNA half molecules. This allowed an unbiased enrichment of the miRNA fraction resulting in a 6-fold increase of mapped miRNA reads and 60% more unique miRNAs detected. Moreover, when comparing miRNA levels in tumor-carrying versus tumor-free mice, we observed a three-fold increase in differentially expressed miRNAs.

MicroRNAs (miRNAs) are a class of small non-coding RNAs that regulate gene expression and play important roles in essential physiological and pathological processes^1^. MiRNAs can be released into circulation and taken up by other cells, altering their gene expression^2^. It has been shown that these cell-free miRNAs are remarkably stable and can be readily detected in the bloodstream^3^,^4^. As miRNAs may exhibit cell-specific expression patterns and as their presence is correlated with specific disease states, such as cancer, numerous studies have investigated the use of circulating miRNAs for disease identification, monitoring and prognostication^5^.

Several measurement technologies are available for assessing relative miRNA abundance: microarray, RT-qPCR and massively parallel sequencing. With differences in reproducibility, accuracy, sensitivity and specificity, the method of choice is strongly dependent on the specific research question^6^. The continuous improvement of cDNA library preparation and sequencing technologies has resulted in an increase of studies evaluating miRNA quantities in blood-derived samples using small RNA sequencing. During the initial read-mapping step of such sequencing experiments, a substantial proportion of reads is often discarded as they map to abundant (and undesirable) RNAs. Further investigation of these discarded reads led to the discovery of new classes of small RNAs derived from well-known small non-coding RNAs like tRNAs, snoRNAs and YRNAs^7^,^8^,^9^,^10^.

In serum, certain tRNA-derived fragments called 5′ tRNA halves appear to be abundantly present, consuming the majority of sequencing reads^11^,^12^,^13^,^14^. 5′ tRNA halves range in size from 30–34 nucleotides and are the product of full length tRNAs being cut in the anti-codon loop by the ribonuclease angiogenin in response to stress^15^,^16^. 5′ tRNA halves can induce assembly of stress granules, inhibit protein translation and their expression in circulation has been found deregulated in cancer^16^,^17^,^18^,^19^,^20^. Despite the fact that 5′ tRNA halves may form an interesting subject of investigation, for sequencing studies examining other small RNA species in serum, their huge overrepresentation embodies an obstacle in reaching the desired experimental output. As 5′ tRNA halves may account for a very large fraction of sequencing reads, they hamper the detection of other small RNA species, like miRNAs. Selective depletion of 5′ tRNA halves could provide an elegant solution for obtaining sufficient sequencing detection power in these types of experiments in the same way ribosomal RNA depletion benefits total RNA sequencing studies^21^.

In this study, we evaluated two complementary approaches for selective depletion of 5′ tRNA halves from murine serum derived RNA based on bead capturing and RNase H cleavage, respectively. Using bead-based depletion, we were able to reduce the 5′ tRNA halves isotypes by more than 95%. This enabled us to increase the number of reads mapping to miRNAs by 6-fold, with 60% more unique miRNAs detected and importantly, with no differential effects on the constitution of the remaining miRNA population. To demonstrate the benefit of performing a 5′ tRNA half depletion, we investigated differentially expressed miRNAs between tumor-free mice and mice carrying orthotopic xenografts. In line with a higher detection rate, the selective depletion of 5′ tRNA halves increased the detectable amount of differentially expressed miRNAs by a factor of three compared to the same non-depleted RNA samples.

## Results

### 5′ tRNA halves are abundantly detected in mouse serum samples using different library preparation methods

First, we evaluated the effect of the small RNA library preparation method on the 5′ tRNA-halves abundance in libraries prepared from serum RNA using three commercially available small RNA library preparation kits. RNA isolated from 100 μl of serum collected from healthy mice was used as input for the preparation of a small RNA library in duplicate and libraries were single end sequenced.

**Figure 1:**
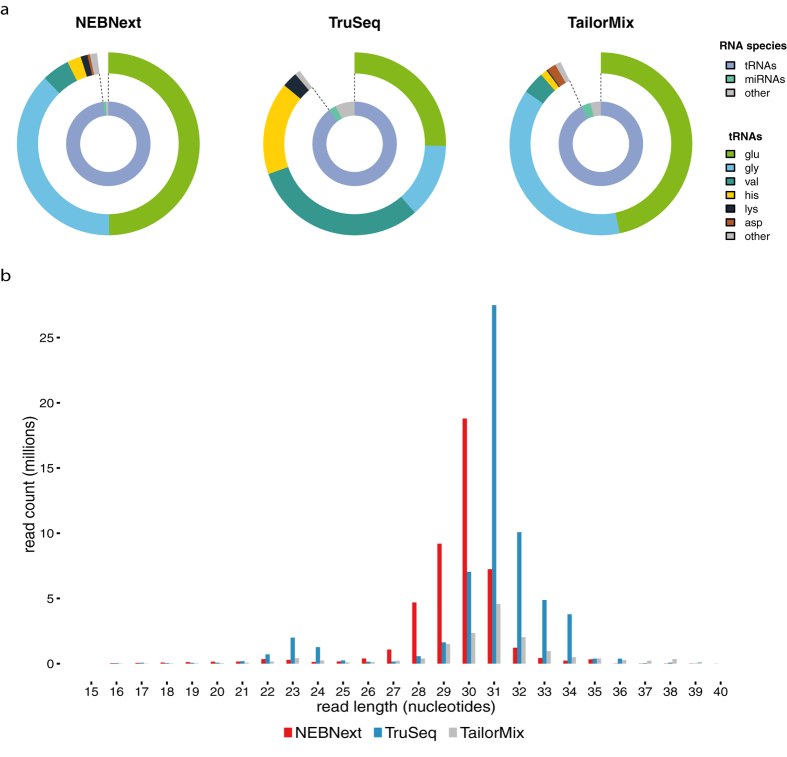
(**a**) Relative abundances of different small RNA species and tRNA-half types in libraries prepared using the NEBNext Multiplex Small RNA library prep kit, the TruSeq Small RNA library prep kit and the TailorMix miRNA sample preparation kit v2. Depicted values represent the amount of small RNA reads as a percentage of the mapped reads. Values represent the mean of two replicates. (**b**) Length distribution of the mapped reads detected in the NEBNext (red), TailorMix (grey) and TruSeq(blue) libraries.

Even though all libraries were sequenced at the same sequencing depth, we observed a clear difference in the number of mapped reads between the different library prep kits. Using the NEBNext kit we obtained between 17.1 and 26.4 million mapped reads, with the TruSeq kit between 10.0 and 12.5 million mapped reads and with the TailorMix kit between 5.3 and 5.5 million mapped reads. Annotation analysis of the mapped sequencing reads revealed that all libraries contained high amounts of reads mapping to tRNA genes, irrespective of library preparation method (Fig. 1a). We found 98.0%, 89.7% and 92.3% of mapped reads annotated as tRNAs for NEBNext, TruSeq and TailorMix libraries respectively. For all three investigated library preparation kits, only a small fraction of mapped reads corresponded to miRNA genes: 0.89%, 2.79% and 3.61% for NEBNext, TruSeq and TailorMix respectively. The distribution of the lengths of the reads displayed a dominant peak at 30–33 nt concordant with the length of tRNA halves (Fig. 1b) with reads mapping to the 5′ end of the tRNA genes.

Investigation of the different types of 5′ tRNA halves present in the libraries revealed that the majority of 5′ tRNA half reads corresponded to just a small subset of tRNAs types. The most abundantly present tRNA types are those with glutamine, glycine, valine and histidine isoacceptors. The relative percentages of these tRNA types vary between the library preparation kits, but their total relative contribution is similar and close to 95% of all tRNA reads (Fig. 1a).

### Effective depletion of 5′ tRNA halves

Next, we evaluated two complementary protocols for 5′ tRNA half depletion for the four tRNA types that we found to be most dominantly present in murine serum: glutamine, glycine, valine and histidine.

For each tRNA half type, different isoacceptors were detected. Comparison of the sequences of the different isotypes showed that it was possible to effectively deplete these fragments using only a limited number of probes for each tRNA type. We designed DNA probes complementary to the most abundantly detected sequences of the four most abundant tRNA half types. In total we designed five probes for depletion of the four most abundant tRNA halves. To interrogate for possible off-target effects, probe sequences were queried against the human and mouse miRNome to identify miRNAs that show perfect complementarity with the probes (Supplementary Table S1). In total five miRNA sequences were found to have complete overlap with either one of the probes.

**Figure 2:**
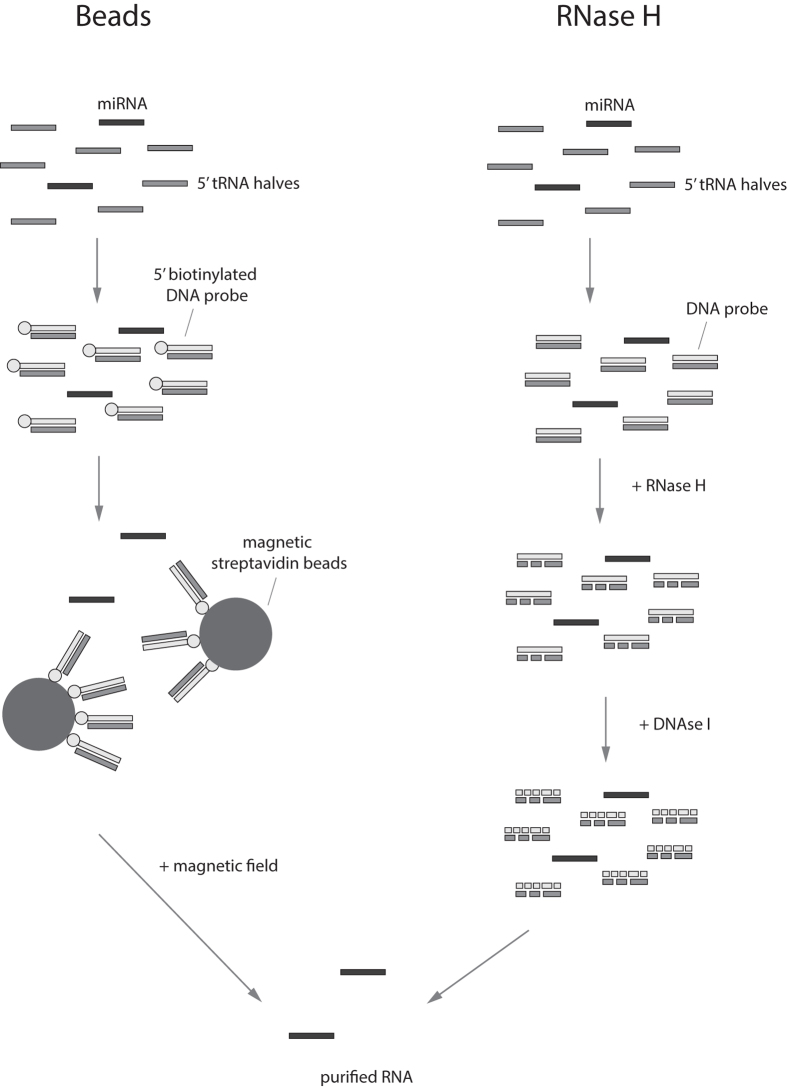
Schematic representation of the two investigated depletion protocols. Beads-based depletion of 5′ tRNA halves involves the use of biotinylated DNA probes with complementarity to the 5′ tRNA half sequences. After DNA/RNA hybridization, probes are bound by magnetic streptavidin beads and immobilized using a magnetic field. Finally, the supernatant, containing purified RNA, is collected and purified by ethanol precipitation. RNase H-based depletion relies on the addition of complementary DNA probes followed by specific cleavage of RNA in DNA/RNA duplexes by RNase H. Remaining DNA probes are then degraded using DNAse I and the depleted RNA purified by ethanol precipitation. See methods section for a more detailed description.

Two complementary approaches were evaluated for their efficacy in depleting 5′ tRNA-halves (Fig. 2). In a first approach, biotinylated DNA probes hybridizing to the target RNA sequences and magnetic streptavidin beads were used to immobilize the DNA-RNA complexes. Using a magnetic field, the beads and bound DNA-RNA complexes were separated after which the supernatant, containing tRNA-depleted RNA, was collected and purified using ethanol precipitation. In the second approach, the addition of DNA probes to the RNA sample and subsequent incubation period were followed by the addition of RNase H, an endonuclease that specifically cleaves the 3′-O-P bond of RNA in a DNA/RNA duplex. After targeted degradation of the tRNA halves, DNA probes were removed by DNase I treatment and the sample was purified by ethanol precipitation.

**Figure 3:**
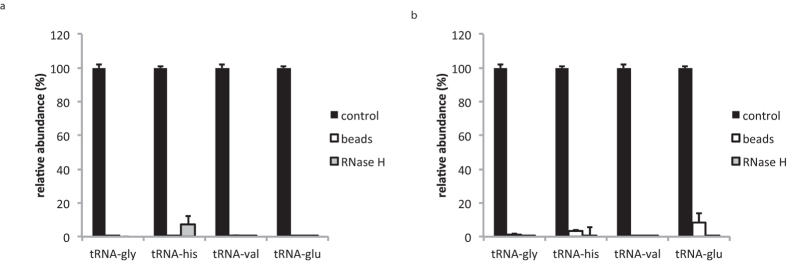
(**a**) tRNA depletion efficiency depicted as relative tRNA abundance values determined by RT-qPCR in non-depleted control samples, samples depleted of target tRNA-halves using RNase H and samples depleted of tRNA-halves using beads. Depicted values represent the mean of two replicates. Error bars represent the standard deviation of two replicates. (**b**) tRNA depletion efficiency depicted as relative tRNA abundance values determined by sequencing of depleted and non-depleted small RNA libraries. Depicted values represent the mean of three replicates. Error bars represent the standard deviation of three replicates.

Both protocols were evaluated on RNA isolated from a serum pool that was collected from healthy SVj mice by cardiac puncture. We assessed the 5′ tRNA half depletion efficiency using RT-qPCR and compared tRNA-half levels between depleted and non-depleted samples (Fig. 3a). For both protocols, very high depletion efficiencies were observed, with more than 99% of the targeted tRNA-types being depleted. To ensure we detected 5′ tRNA halves, and not their full-length tRNA counterparts, we evaluated the size of the PCR amplicons by capillary electrophoresis (LabChip, Caliper) and found that amplicon sizes correspond to 5′ tRNA half fragments (Supplementary Figure S1).

To assess whether the depletion protocols could affect miRNA expression values, we determined the abundance levels of mmu-miR-16-5p (MIMAT0000527, miRBase v18), a miRNA that is reported to be highly and stably expressed in mouse serum, in depleted and control samples^22^. Both depletion protocols were found to have an impact on miR-16 expression, with a 2-fold and 20-fold reduction in miR-16 expression levels for the beads-based depletion and RNase H-based depletion, respectively (Supplementary Figure S2). We did not observe a reduction in miRTC levels, a reverse transcriptase control assay, excluding the possibility of PCR or reverse transcription inhibition. Even with a reduction in absolute miRNA quantities, a depletion protocol can still prove to be effective, given that the relative quantities as compared to the population of 5′ tRNA halves are substantially increased and that effect is similar for all miRNAs.

### Effective depletion of tRNA halves for high-throughput sequencing of miRNAs

To evaluate the efficacy of the described protocols on small RNA sequencing, we performed the two 5′ tRNA halves depletion protocols on total RNA isolated from a serum pool collected from healthy mice. Each RNA sample was divided into three and the resulting fractions were either depleted using beads or RNase H, or used as a control. The experiment was performed in triplicate. Upon depletion, libraries were prepared using TruSeq Small RNA library preparation kit (given the higher number of mapped reads and miRNA reads as compared to the NEBNext and TailorMix kits). Samples were sequenced as described above.

**Figure 4:**
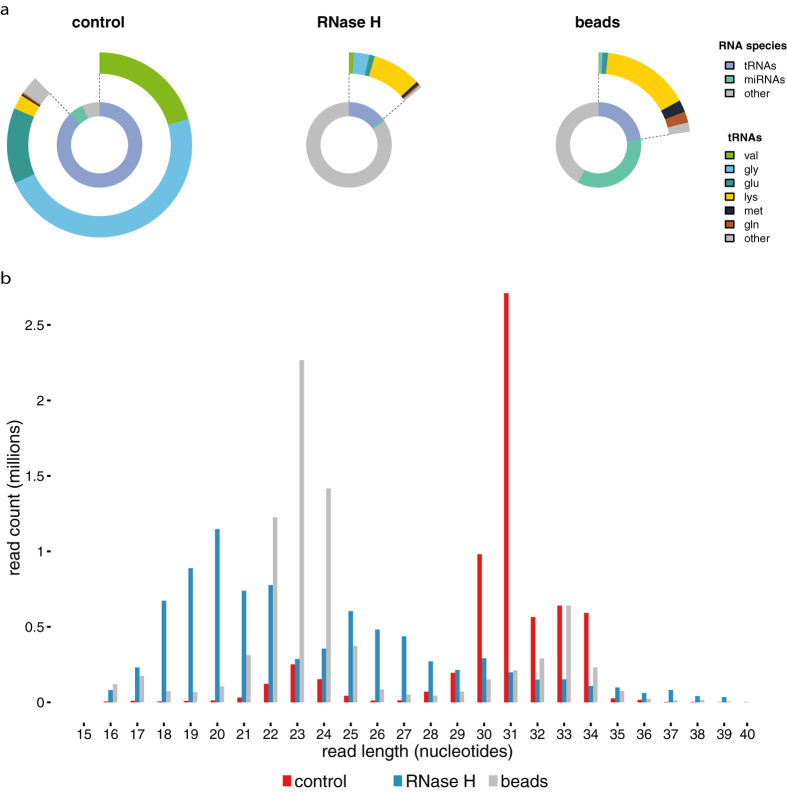
(**a**) Relative abundances of different small RNA species and tRNA-half types in depleted and non-depleted libraries. Depicted values represent the amount of small RNA reads as a percentage of the mapped reads. Values represent the mean of two replicates. (**b**) Length distribution of the mapped reads detected in libraries depleted using beads (grey), libraries depleted using RNase H (blue) and non-depleted libraries (red).

We observed a clear difference in the average number of mapped reads, 4.63 × 10^6^, 1.77 × 10^5^ and 5.46 × 10^6^ for control, RNase H-depleted and beads-depleted samples, respectively. Comparison of the relative abundance of the different RNA species revealed a pronounced reduction in the fraction of detected 5′ tRNA halves in depleted samples as compared to non-depleted samples (Fig. 3b), with depletion efficiencies similar as previously measured by RT-qPCR (Fig. 3a). In non-depleted RNA samples, 87.79% of mapped reads were mapping to tRNA halves, 5.65% to miRNAs and 6.55% to other small RNA fragments (Fig. 4a). In RNA samples depleted using RNase H, 14.6% of mapped reads were assigned to 5′ tRNA halves, only 0.9% to miRNA and 84.0% to other small RNA fragments (Fig. 4a). For RNA samples depleted using beads, we observed that 23% of mapped reads map to 5′ tRNAs halves, 35% to miRNAs and 42% map to other small RNA fragments (Fig. 4a). These results are illustrated in a frequency histogram of the RNA length distribution. In samples depleted by beads, most RNA molecules have a length between 20–25 nucleotides. In samples depleted using RNase H, the distribution of RNA lengths is spread out between 18 and 30 nucleotides (Fig. 4b).

**Figure 5:**
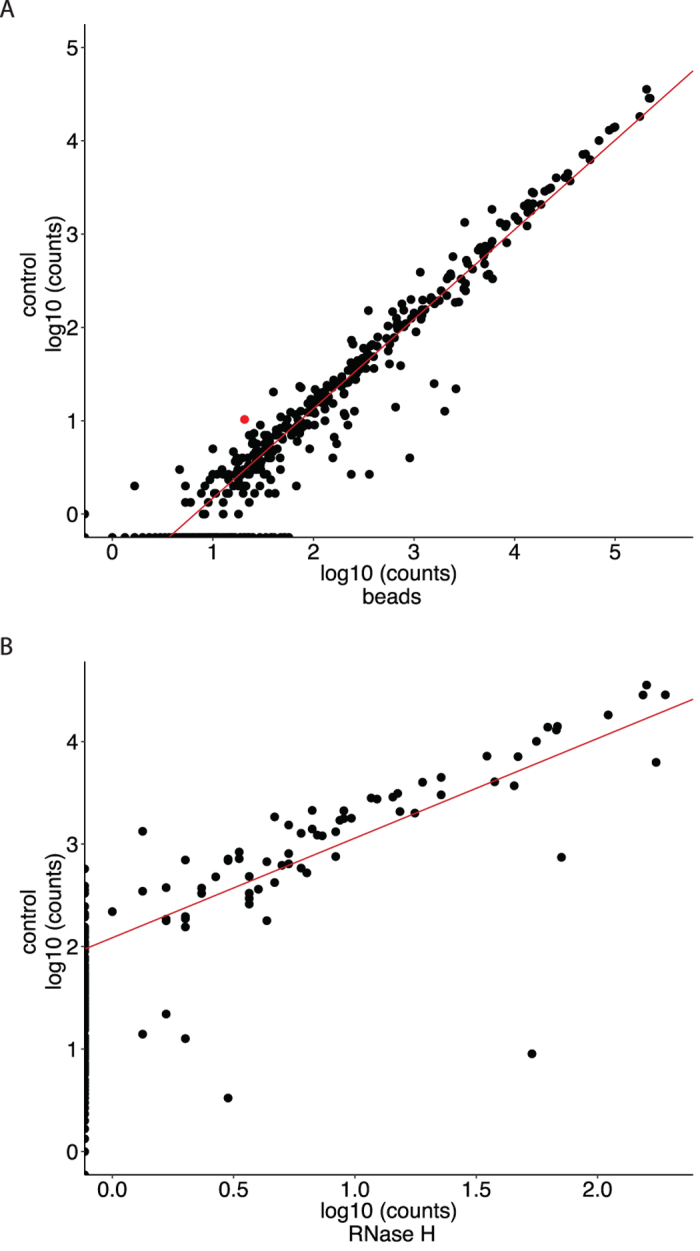
Correlation of miRNA expression values in control samples versus depleted samples using beads (**A**) and RNase H (**B**). Expression values are depicted as log10 of the non-normalized counts. Depicted values represent the mean of three replicates. Red dots represent miRNAs that show (partial) complementarity with one of the used probes.

Taking into account the library sizes, the observed reduction of tRNA-halves using beads results in a 6-fold increase in mapped miRNA reads (Table 1). In addition, almost 50% more unique miRNAs are detected, 52.17 unique miRNAs per million mapped reads in control samples against 75.82 unique miRNAs per million mapped reads in depleted samples or, in total, 240 detected miRNAs in control samples compared to 417 in depleted samples (Table 1). In contrast, depletion using RNase H resulted in a strong reduction in both miRNA reads and detected miRNAs as compared to the control samples, with a total of 40 detected miRNAs in depleted samples (Table 1). Finally, we assessed whether the depletion protocols affect miRNA expression values, as it cannot be excluded a priori that the probes also bind miRNAs. To this purpose a correlation analysis of miRNA expression levels in depleted against non-depleted control samples was performed. For beads we observed a high correlation (Pearson r = 0.99, n = 310) between depleted and control samples (Fig. 5A), including miRNAs showing (partial) complementarity with the used probes. For RNase H the correlation was less strong (Pearson r = 0.87, n = 69) (Fig. 5B). The bead-based depletion protocol does not considerably affect relative abundances of individual miRNAs.

### Differential miRNA expression upon tRNA halves depletion of low serum volumes

To investigate the effectiveness of performing 5′ tRNA half depletion in a real miRNA quantification experiment, we evaluated the association of tumor burden on circulating miRNAs present in mouse serum. Three nude mice were orthotopically injected with SH-SY5Y human neuroblastoma cells. Serum samples were collected one week before cell injection and two weeks after injection, when mice had palpable tumors. We performed 5′ tRNA half depletion using the beads protocol (probes sequences in Supplementary Table S3) as it performed remarkably better than the RNase H protocol. Small RNA libraries were prepared using the TruSeq small RNA library prep kit and sequenced as described above.

**Figure 6:**
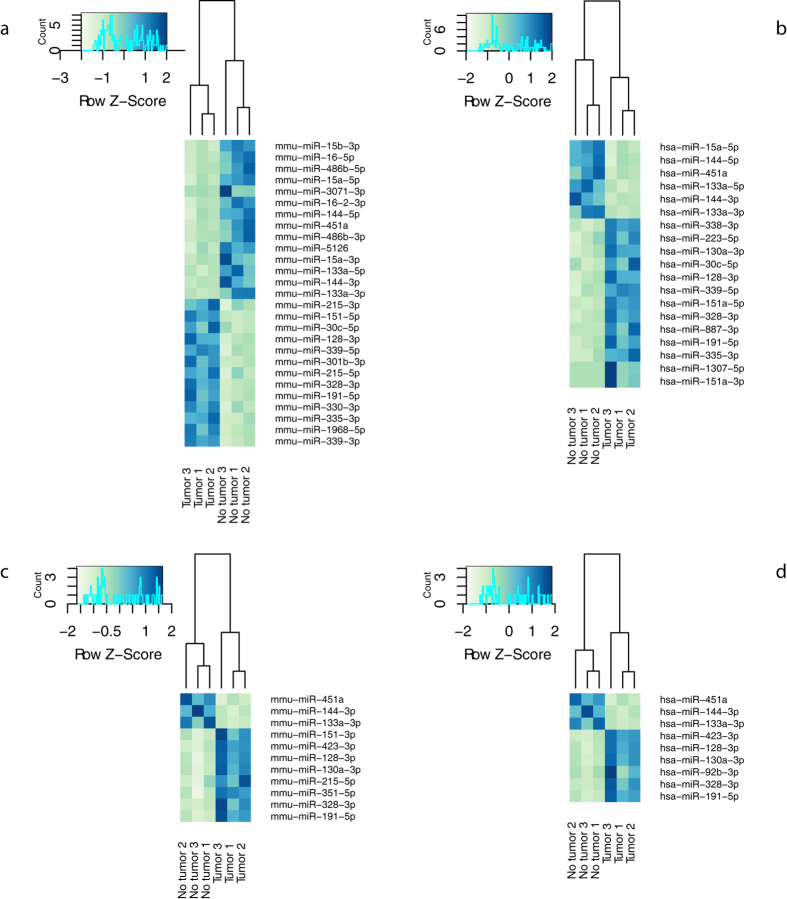
Hierarchical clustering of samples based on significantly differentially expressed miRNAs in serum collected before tumor cell injection and after tumor manifestation. Color areas indicate z-scores across all samples based on normalized miRNA expression values. (**a**) murine miRNAs in RNA depleted from 5′ tRNA halves; (**b**) human miRNAs in RNA depleted from 5′ tRNA halves. (**c**) murine miRNAs in non-depleted RNA. (**d**) human miRNAs in non-depleted RNA. miRNA names are according to miRBase v21 annotation.

When applying an expression cut-off of 4 counts, we detect on average 261 murine miRNAs, 123 human miRNAs and 329 miRNAs with complete conservation between mouse and human in depleted samples. In non-depleted control samples we detect 101 murine miRNAs, 25 human miRNAs and 137 miRNAs with complete conservation between the two species. When comparing miRNA expression values in tumor-bearing mice with tumor-free mice and applying a fold change cut-off of 2, we find in total 34 differentially expressed miRNA (18 upregulated and 16 downregulated; p-adjusted < 0.05) in depleted samples (Fig. 6). Of the differentially expressed miRNAs six are human miRNAs, 13 are murine miRNAs and 15 miRNAs have complete conservation between mouse and human (Supplementary Table S4 & Fig. 6a,b). In non-depleted samples, we find in total only 12 differentially expressed miRNAs (9 upregulated and 3 downregulated, p-adjusted < 0.05) (Supplementary Table S4 & Fig. 6c,d). Out of these, four are murine miRNAs and eight miRNAs have complete conservation between mouse and human. Of note, several miRNAs that we found differentially expressed have been described before in the context of neuroblastoma. Hsa-miR-15a-5p, hsa-miR-16-5p, hsa-miR-30c, hsa-miR-451a, hsa-miR-191-5p, hsa-miR-486-5p and hsa-miR-335-3p for instance have been described to be aberrantly expressed in metastatic neuroblastoma tumors^23^. Some of these, like hsa-miR-30c or hsa-miR-486-5p were only found differentially expressed in depleted samples, further underlining the benefit of performing 5′ tRNA halves depletion. In addition, only in depleted samples, sensitivity was high enough to detect differentially expressed human-specific miRNAs like hsa-miR-144-5p, a miRNA that is reported to be overexpressed in human neuroblastoma cells^24^.

## Discussion

Low RNA concentration due to its low abundance in serum poses a critical obstacle for the exact quantification of serum-derived miRNAs, especially for sequencing-based technologies. There have been several efforts to improve discrete steps of the miRNA quantification process in order to enhance the efficacy of miRNA detection in these types of samples. As such, improvement of pre-analytical parameters such as storage conditions and serum preparation, optimization of the RNA extraction process and advancements in library preparation protocols were found to improve miRNA detection performance^25^,^26^,^27^,^28^,^29^. Despite these efforts, the efficiency of small RNA sequencing of serum samples is still very low. Given the high abundance of 5′ tRNA halves in small RNA libraries prepared from serum samples that potently hinders the detection of other small RNA species, targeted depletion of these 5′ tRNA halves could prove highly efficient.

In this study we optimized and compared two protocols for the selective depletion of 5′ tRNA halves in RNA isolated from murine serum samples of which only one protocol was successful in targeted depletion of 5′ tRNAs halves. Using a protocol consisting of target capture by biotinylated DNA probes followed by immobilization using magnetic streptavidin beads, we were able to successfully deplete up to 99% of the targeted 5′ tRNA halves in RNA samples. Depletion of 5′ tRNA halves in small RNA libraries resulted in a more than 6-fold increase in mapped miRNA reads and 60% more detected mature miRNAs when performing small RNA sequencing on murine serum samples. Importantly, we observed no considerable effects on the relative expression values of miRNAs. Moreover, using 5′ tRNA halves depletion we were able to detect 3 times more differentially expressed miRNAs when comparing serum from mice carrying orthotopically engrafted tumors with serum from healthy control mice. We detected 34 differentially expressed miRNAs of which six are unique to humans, 13 unique to mice and 15 have complete conservation between the two species. Especially for such applications, in which the miRNA sequence itself can carry information about its cellular origin, sequencing is very well suited because of its exquisite specificity when strict mapping is applied. In contrast, RNase H-based depletion did not prove highly effective. It could be that cleaved tRNA half fragments are still being processed during library preparation as RNAse H can yield polished, easily ligated RNA ends.

It is straightforward to further increase the efficacy of 5′ tRNA halves depletion by including additional capture probes that target other 5′ tRNA isoacceptors not targeted in this study. It has been reported that the expression of circulating 5′ tRNA halves derived from specific tRNA isoacceptors can change as a result of experimental conditions like aging and calorie restriction^11^. It is not yet clear whether the same types of 5′ tRNA halves are present in all murine serum samples or whether their relative abundance depends on the mouse strain or other experimental and physiological conditions. In the mouse strains we studied, we found that circulating 5′ tRNA halves derived from a limited number of tRNA isoacceptors to be most dominantly present. Moreover, these were the same tRNA-types as were previously reported for murine serum^11^. These results suggest that it could be sufficient to design probes targeting only a limited set of 5′ tRNA-half isotypes to allow for effective depletion across a wide range of murine serum samples. More sequencing studies on mouse serum are needed to confirm these assumptions. Finally, we anticipate that our capture based depletion protocol could also be used for depletion of other abundant sequences present in any type of RNA samples with tailored probe sets.

## Methods

### Blood collection and serum preparation

Healthy male and female 129/SVj mice, with ages varying between 2 to 9 months, were sedated using tribromoethanol at a dose of 250 mg/kg injected intraperitoneally. 500 μl to 1 ml of blood per mouse was collected in Safe-Lock micro test tubes (Eppendorf) by cardiac puncture using a 0.45 × 12 mm needle after which mice were sacrificed by cervical dislocation. For NCr nude mice (Taconic Farms), 100 μl blood was collected by puncture of the jugular vein using a 4 mm lancet and collected in a BD Vacutainer collection tube with a gel separator (BD). All blood samples clotted at room temperature for 45 min. Clotted blood samples from healthy SVj mice were centrifuged at 1900 g for 10 min at 4 °C using a swinging bucket rotor. The supernatant, containing the serum, was collected and centrifuged again at 16000 g for 10 min at 4 °C in a fixed angle rotor to pellet cellular nucleic acids attached to cell debris. The supernatant was collected and stored at −80 °C. Clotted blood samples of nude mice were centrifuged at 2000 g for 15 minutes at 4 °C in a fixed angle rotor. The supernatant was collected and stored at −80 °C. For all serum samples, the degree of hemolysis was determined by measuring levels of free hemoglobin by spectral analysis. Absorbance peaks at 414, 541 and 576 nm are indicative of free hemoglobin^30^. Experimental protocols were approved by the Ethical committee of University Ghent and Baylor College of Medicine and carried out in accordance with the relevant guidelines and regulations.

### Orthotopic xenograft model

Orthotopic neuroblastoma xenografts were generated in four to six week old female athymic immunodeficient NCr nude mice as previously described^31^. Briefly, 1 × 10^6^ human SH-SY5Y neuroblastoma cells were surgically implanted in beneath the renal capsule. This model closely resembles the growth characteristics of primary neuroblastoma arising from the para-adrenal location in humans^32^. All experimental protocols were approved by the Ethical committee of Baylor College of Medicine and carried out in accordance with the relevant guidelines and regulations.

### Total RNA isolation

RNA was isolated using the miRNeasy serum/plasma kit (Qiagen) according to the manufacturer’s instructions. For the final experiment, 50, 100 or 200 μl of serum was used as input and total RNA was eluted in 12 μl of RNAse-free water.

RNA used for the evaluation of the different library preparation kits was isolated from 100 μl of serum collected from individual SVj mice and eluted in 12 μl of RNAse-free water. For RNA isolation of samples used for the assessment of effective tRNA depletion by sequencing, serum samples from 3 individual SVj mice were pooled and 200 μl aliquots of the serum pool were used as input for RNA isolation and total RNA was eluted in 12 μl of RNAse-free water. After elution from the miRNeasy column, RNA samples used for the assessment of tRNA depletion by sequencing were diluted to a final volume of 38 μl and distributed in 12 μl aliquots. RNA used for differential expression analysis was isolated from 50 μl of serum that was collected from Ncr nude mice and eluted in 12 μl of RNAse-free water.

### tRNA fragment depletion

5′ biotinylated DNA probes were designed for four 5′ tRNA halves species (Supplementary Table S1) and ordered from IDT. To evaluate for off-target effects, probe sequences were queried against the human and mouse miRNome to identify miRNAs that show 100% complementarity with the probes (Supplementary Table S1).

For tRNA halves depletion using beads, 12 μl of RNA, 15 μl of 2x hybridization buffer (Supplementary file S1), 1 μl of tRNA halves capture probes (at a final reaction concentration of 0.5 μM for each probe) and 2 μl of RNase-free water were incubated at 80 °C for 2 min to denature the RNA. The mixture was slow-cooled to 22 °C (at 0.1 °C/min) and placed on ice to allow for efficient hybridization. Dynabeads Myone Streptavidin C1 (Life Technologies) were washed 3 times using a washing buffer (Supplementary file S1). After washing, beads were prepared for RNA manipulation by washing twice with solution A and once with solution B (Supplementary file S1) and suspended in 2x washing buffer to a final concentration of 5 μg/μl. Next, 30 μl of sample was added to 30 μl of beads and the mixture was incubated for 10 min at room temperature with gentle mixing. The mixture was then placed on a magnet for 2 min after which the supernatant, containing the depleted RNA, was collected. The depleted RNA was purified by ethanol precipitation and suspended in 7.5 μl RNase-free water (Supplementary file S1).

For tRNA halves depletion using RNAse H, 2 μl of 7.5x hybridization buffer (Supplementary file S1) was combined with 1 μl of biotinylated probes (final probe concentration of 0.5 μM/probe) and added to 12 μl of RNA. Hybridization was performed under the same conditions as described above. After hybridization, 1 μl of RNase H (10,000 U/ml, Epicentre), 2 μl of 10x RNase H reaction buffer (Supplementary file S1) and 2 μl of 0.1 mM DTT was added to the sample and the mixture was incubated at 37 °C for 30 min. After RNase H digestion, 5 μl of 10x DNase I reaction buffer (Supplementary file S1), 2.5 μl DNAse I (2500 U/ml, Life Technologies) and 22.5 μl RNase-free water was added to the digested sample and the mixture was incubated at 37 °C for 30 min. The depleted RNA was purified using ethanol precipitation and resuspended in 7.5 μl RNase-free water.

### RT-qPCR quantification of tRNA fragments

For reverse transcription we used the miScript II RT kit (Qiagen) with HiFlex buffer according to manufacturer’s instructions but with 1.5 μl of input RNA. After reverse transcription, the resulting cDNA was diluted 22-fold by adding 210 μl nuclease-free water to 10 μl of RT product.

For detection of tRNA-halves by RT-qPCR the miScript SYBR green PCR kit (Qiagen) was used with custom designed primer assays (Supplementary Table S2). The RT-qPCR was performed in duplicate in a 10 μl reaction volume using 5 μl of 2x Quantitect SYBR Green PCR Master mix, 1 μl of 10x miSCript universal primer, 1 μl target specific primer (5 μM), 1 μl of RNase-free water and 2 μl of diluted cDNA were combined. PCR amplification was performed on a CFX384 real-time PCR detection system (Bio-Rad). Enzyme activation at 95 °C for 15 min followed by 45 cycles of 94 °C for 15 s, 55 °C for 30 s and 70 °C for 30 s. No template samples were included as negative control. Cq values were determined by the regression method (CFX manager v3.1, Bio-Rad) and the depletion efficiency was determined by the ΔCq method assuming 100% PCR efficiency. A reverse transcriptase control assay (miRTC, Qiagen) was included to evaluate RT inhibition.

### Small RNA sequencing

For Small RNA library preparation we used the TailorMix miRNA sample preparation kit v2 (Seqmatic), the NEBNext Multiplex Small RNA library prep kit (New England Biolabs) or the TruSeq small RNA library preparation kit v2 (Illumina) following manufacturer’s instructions with small modifications (Supplementary file S1). Adaptor sequences can be found in Supplementary Table S5. After PCR amplification, quality of libraries was assessed using a high sensitivity DNA kit on a Bioanalyzer (Agilent) according to manufacturer’s instructions. Size selection was performed using 3% agarose dye-free marker H cassettes on a Pippin Prep (Sage Science) following manufacturer’s instructions with a specified collection size range of 125–153 bp. Libraries were further purified and concentrated by ethanol precipitation, resuspended in 10 μl of 10 mM tris-HCl (pH = 8.5) and quantified using qPCR (see further). Based on the qPCR results, equimolar library pools were prepared, quality was assessed as described above and the library was further diluted to 4 nM using 10 mM tris-HCl (pH = 8.5). The pooled library was then sequenced at a final concentration of 1.2 pM on a NextSeq 500 using a mid or high output v2 kit (single-end, 75 cycles, Illumina).

For RT-qPCR quantification of sequencing libraries, 1 μl of EtOH purified library was diluted 1:100 000. 2.5 μl of SsoAdvanced universal SYBR green supermix (Bio-Rad Laboratories) and 0.25 μl of each primer (5 μM; Supplementary Table S2) were combined with 2 μl of diluted library. Reactions were set up in duplicate and performed in a LightCycler 480 (Roche) using the following protocol: enzyme activation at 95 °C for 15 min, followed by 44 cycles of 95 °C for 5 s, 60 °C for 30 s and 72 °C for 1 s.

### Sequencing data analysis

For the quantification of small RNAs Biogazelle’s dedicated small RNA seq pipeline was used (part of Cobra), as publically available alternatives we advice the use of miRDeep2 and miRExpress. Adaptor trimming was performed using Cutadapt v1.8.1, reads shorter than 15 bp and those in which no adaptor was found, were discarded. For quality control the FASTX-Toolkit (v0.0.14) was used, a minimum quality score of 20 in at least 80% of bases was applied as a cutoff. The reads were mapped with Bowtie (v1.1.2) without allowing for mismatches. Mapped reads were annotated by matching genomic coordinates of each read with genomic locations of miRNAs (obtained from miRBase, v20) and other small RNAs (obtained from UCSC (human: GRCh37/hg19; mouse: GRCm38/mm10) and Ensembl, v84). Raw and processed sequencing data have been submitted to the GEO database (GSE88915). Further data analysis was performed using RStudio (v0.99.486, RStudio). Normalization of miRNA counts and differential expression analysis was performed using the R package DESeq2 (v1.8.2). The DESeq2 result tables are available in Supplementary dataset S1.

## Additional Information

**How to cite this article**: Van Goethem, A. *et al*. Depletion of tRNA-halves enables effective small RNA sequencing of low-input murine serum samples. *Sci. Rep.* **6**, 37876; doi: 10.1038/srep37876 (2016).

**Publisher's note:** Springer Nature remains neutral with regard to jurisdictional claims in published maps and institutional affiliations.

## Supplementary Material

Supplementary Files

Supplementary Dataset 1

## Figures and Tables

**Table 1:** Sequencing output of non-depleted control samples, samples depleted using RNase H and samples depleted using beads.

	control	RNase H	beads
mapped reads (millions)	4.6 +/− 0.3	0.18 +/− 0.04	5.5 +/− 0.1
total reads (millions)	7.5 +/− 0.5	8.5 +/− 1.0	8.9 +/− 0.3
mapped miRNAs reads (millions)	0.26 +/− 0.04	0.0017 +/− 0.0002	1.91 +/− 0.05
unique miRNAs	240 +/− 9	40 +/− 5	417 +/− 6

Depicted values represent the mean of three samples +/− the standard error.
