# Supplementary material for: Depletion of tRNA-halves enables effective small RNA sequencing of low-input murine serum samples
Source: Sci Rep. 2016 Nov 30;6:37876. doi: 10.1038/srep37876 (PMC5129013; doi:10.1038/srep37876)
Supplement: Supplementary Files [file srep37876-s1.pdf]

Depletion of tRNA-halves enables effective small RNA sequencing of low-input murine serum samples

Alan Van Goethem, Nurten Yigit, Celine Everaert, Myrthala Moreno-Smith, Liselot M. Mus, Eveline Barbieri, Frank Speleman, Pieter Mestdagh, Jason Shohet, Tom Van Maerken, Jo Vandesompele

Supplementary figure 1:

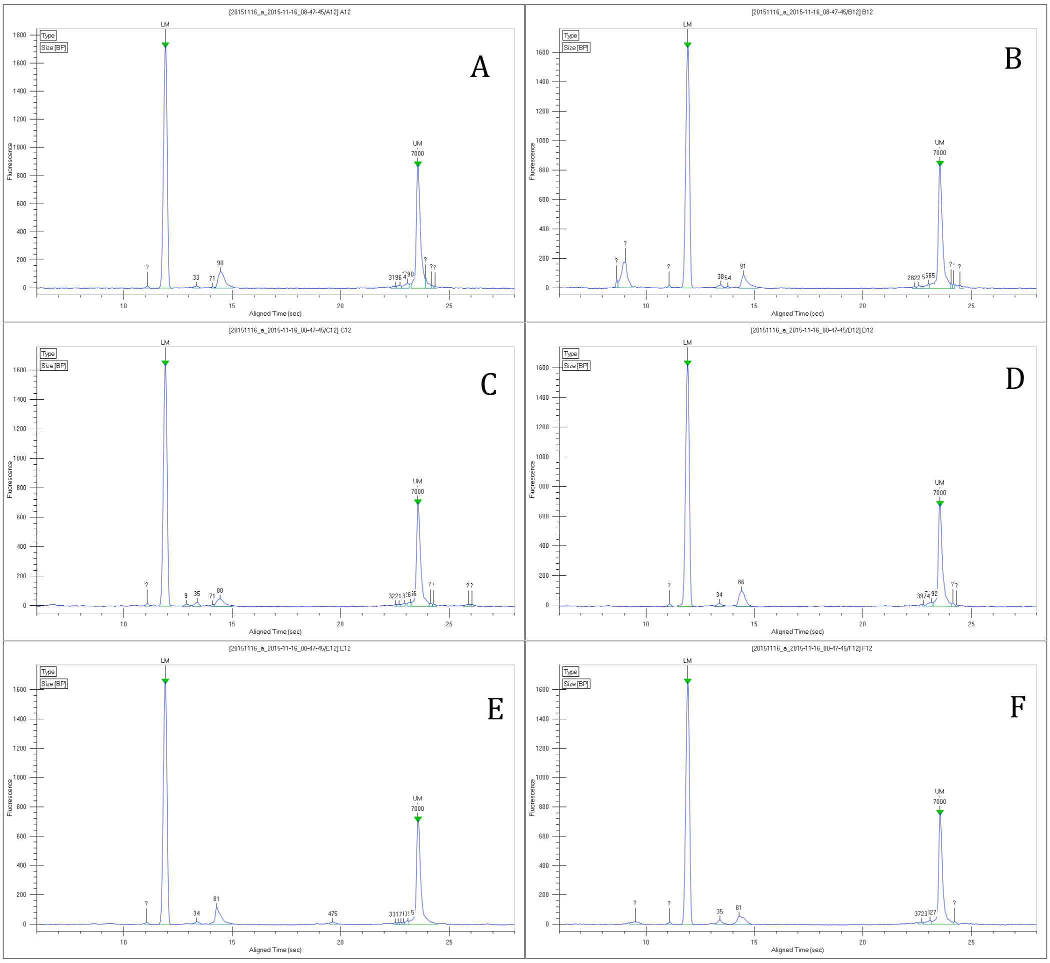

supplementary figure 2: PCR amplicon size evaluation by capillary gel electrophoresis. A: tRNA-gly; B: tRNA-his ; C: tRNA-val ; D: tNRA-glu; E: tRNA-gly; F: tRNA-glu.

Supplementary figure 2:

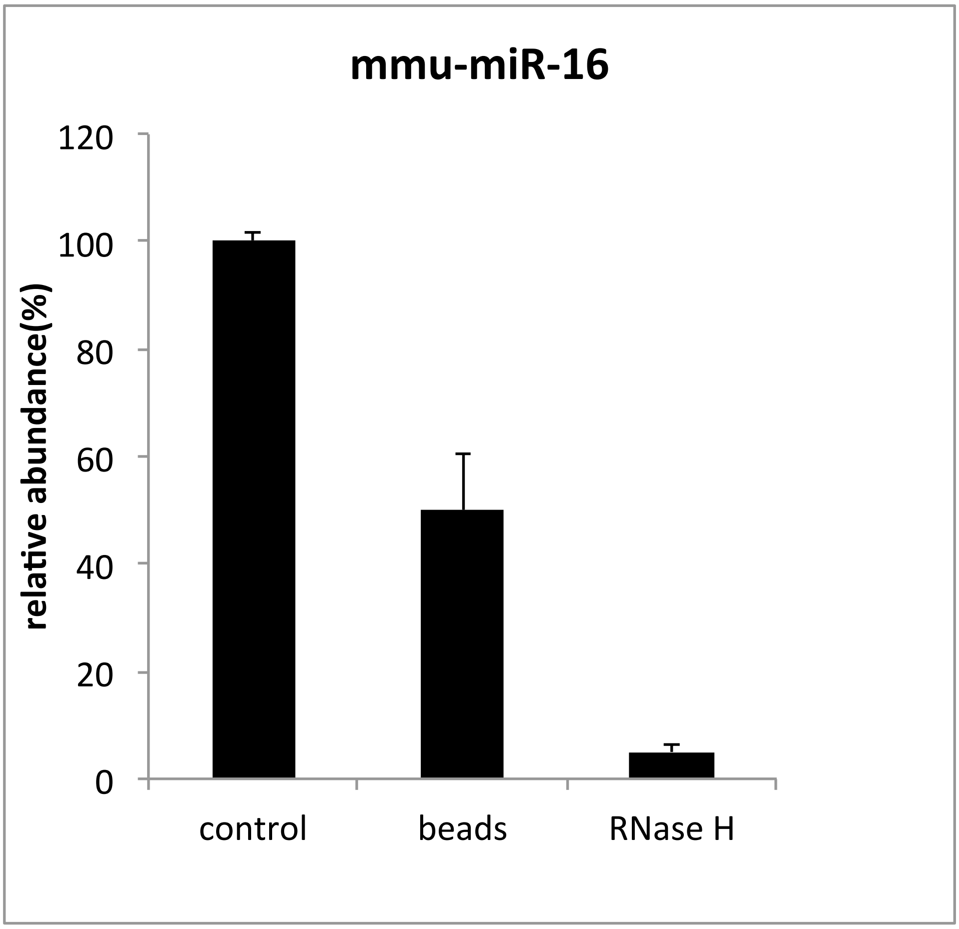

supplementary figure 3: Relative abundance of mmu-miR-16 in beads depleted and RNase H depleted murine serum samples, compared to non-depleted control sample.

Supplementary table 1:

5' tRNA half	RNA sequence	probe sequence	miRNA matches (miRBase v21)
tRNA-glu	UCCCUGGUGGUCUAGUGGUUAGGAUUCGGCG	CGCCGAATCCTAACCACTAGACCACCAGGGA	hsa-miR-4259 (MIMAT0016880)
tRNA-gly	GCAUGGGUGGUUCAGUGGUAGAAUUCUCGC	GCGAGAATTCTACCACTGAACCACCCATGC	mmu-miR-183-5p (MIMAT0000212), hsa-miR-183-5p (MIMAT0000261), mmu-miR-7046-3p (MIMAT0027997)
tRNA-gly	GCAUUGGUGGUUCAGUGGUAGAAUUCUCGCC	GGCGAGAATTCTACCACTGAACCACCAATGC	mmu-miR-183-5p (MIMAT0000212), hsa-miR-183-5p (MIMAT0000261)
tRNA-his	GGCCGUGAUCGUAUAGUGGUUAGUACUCUGC	GCAGAGTACTAACCACTATACGATCACGGCC	no miRNA match
tRNA-val	GUUUCCGUAGUGUAGUGGUUAUCACGUUCGCCU	AGGCGAACGTGATAACCACTACACTACGGAAAC	mmu-miR-471-5p (MIMAT0002112), hsa-miR-3182(MIMAT0015062)

supplementary table 1: Probe sequences used for depletion of serum-derived RNA samples and miRNAs that show (partial) complementarity with the used probes.

Supplementary table 2:

	primer sequence
tRNA-gly	GCTTTGGTGGTTCAGTGGTAG
tRNA-his	GGCCGTGATCGTATAGTGGT
tRNA-val	TCCGTAGTGTAGTGGTTATCAC
tRNA-glu	TCCCTGGTGGTCTAGTGGTT
tRNA-gly	GCATTGGTGGTTCAGTGGTAG
tRNA-glu	TCCCTGTGGTCTAGTGGTTGA
adaptors TruSeq forward	AATGATACGGCGACCACCGA
adaptors TruSeq reverse	CAAGCAGAAGACGGCATACGA

supplementary table 2: Primer sequences used for the detection of 5' tRNA halves and the quantification of small RNA sequencing libraries using RT-qPCR.

Supplementary table 3:

tRNA	RNA sequence	probe sequence	miRNA matches (miRBase v21)
tRNA-glu	UCCCUGGUGGUCUAGUGGUUAGGAUUCGGCG	CGCCGAATCCTAACCACTAGACCACCAGGGA	hsa-miR-4259 (MIMAT0016880)
tRNA-gly	GCAUGGGUGGUUCAGUGGUAGAAUUCUCGC	GCGAGAATTCTACCACTGAACCACCCATGC	mmu-miR-183-5p (MIMAT0000212), hsa-miR-183-5p (MIMAT0000261), mmu-miR-7046-3p (MIMAT0027997)
tRNA-gly	GCAUUGGUGGUUCAGUGGUAGAAUUCUCGCC	GGCGAGAATTCTACCACTGAACCACCAATGC	mmu-miR-183-5p (MIMAT0000212), hsa-miR-183-5p (MIMAT0000261)
tRNA-his	GGCCGUGAUCGUAUAGUGGUUAGUACUCUGC	GCAGAGTACTAACCACTATACGATCACGGCC	no miRNA match
tRNA-val	GUUUCCGUAGUGUAGUGGUUAUCACGUUCGCCU	AGGCGAACGTGATAACCACTACACTACGGAAAC	mmu-miR-471-5p (MIMAT0002112), hsa-miR-3182(MIMAT0015062)
tRNA-lys	GCCCGGCUAGCUCAGUCGGUAGAGCAUGAGACU	AGTCTCATGCTCTACCGACTGAGCTAGCCGGGC	hsa-miR-6078 (MIMAT0023703), mmu-miR-5106 (MIMAT0020613)
tRNA-lys	GUCCCAUGCUCUACCGACUGAGCUAGCCGGGC	GCCCGGCTAGCTCAGTCGGTAGAGCATGGGAC	hsa-miR-6078 (MIMAT0023703), mmu-miR-5106 (MIMAT0020613)
tRNA-met	AGCAGAGUGGCGCAGCGGAAGCGUGCUGGGC	GCCCAGCACGCTTCCGCTGCGCCACTCTGCT	no miRNA match
tRNA-gln	GGUUCCAUGGUGUAAUGGUUAGCACUCUGGACUC	GAGTCCAGAGTGCTAACCATTACACCATGGAACC	mmu-miR-6356 (MIMAT0025099), mmu-miR-759 (MIMAT0003897), hsa-miR-759 (MIMAT0010497)
tRNA-arg	GACCCAGUGGCCUAAUGGAUAAGGCAUCAGCCU	AGGCTGATGCCTTATCCATTAGGCCACTGGGTC	no miRNA match
tRNA-pro	GGCUCGUUGGUCUAGGGGUAUGAUUCUC	GAGAATCATACCCCTAGACCAACGAGCC	hsa-miR-4448 (MIMAT0018967)

supplementary table 3: Depletion probe sequences used for comparing miRNA expression levels in tumor-carrying versus tumor-free mice and miRNAs that show (partial) complementarity with the used probes.

Supplementary table 4:

Depleted samples					
human miRNA ID	human miRNA	murine miRNA ID	murine miRNA	FC	sequence
MIMAT0000062	hsa-let-7a-5p	MIMAT0000521	mmu-let-7a-5p	0.56	UGAGGUAGUAGGUUGUAUAGUU
MIMAT0000068	hsa-miR-15a-5p	MIMAT0000526	mmu-miR-15a-5p	0.46	UAGCAGCACAUAAUGGUUUGUG
MIMAT0000069	hsa-miR-16-5p	MIMAT0000527	mmu-miR-16-5p	0.48	UAGCAGCACGUAAAUAUUGGCG
MIMAT0000081	hsa-miR-25-3p	MIMAT0000652	mmu-miR-25-3p	0.51	CAUUGCACUUGUCUCGGUCUGA
MIMAT0000084	hsa-miR-27a-3p	MIMAT0000537	mmu-miR-27a-3p	1.54	UUCACAGUGGCUAAGUUCCGC
MIMAT0000087	hsa-miR-30a-5p	MIMAT0000128	mmu-miR-30a-5p	0.52	UGUAAACAUCCUCGACUGGAAG
MIMAT0000093	hsa-miR-93-5p	MIMAT0000540	mmu-miR-93-5p	0.57	CAAAGUGCUGUUCGUGCAGGUAG
MIMAT0000099	hsa-miR-101-3p	MIMAT0000133	mmu-miR-101a-3p	0.54	UACAGUACUGUGAUAACUGAA
MIMAT0000244	hsa-miR-30c-5p	MIMAT0000514	mmu-miR-30c-5p	2.05	UGUAAACAUCCUACACUCUCAGC
MIMAT0000424	hsa-miR-128-3p	MIMAT0000140	mmu-miR-128-3p	2.13	UCACAGUGAACCGGUCUCUUU
MIMAT0000425	hsa-miR-130a-3p	MIMAT0000141	mmu-miR-130a-3p	1.99	CAGUGCAAUGUUAAAAGGGCAU
MIMAT0000427	hsa-miR-133a-3p	MIMAT0000145	mmu-miR-133a-3p	0.33	UUUGGUCCCCUUCAACCAGCUG
MIMAT0000435	hsa-miR-143-3p	MIMAT0000247	mmu-miR-143-3p	0.58	UGAGAUGAAGCACUGUAGCUC
MIMAT0000436	hsa-miR-144-3p	MIMAT0000156	mmu-miR-144-3p	0.33	UACAGUAUAGAUGAUGUACU
MIMAT0000440	hsa-miR-191-5p	MIMAT0000221	mmu-miR-191-5p	2.32	CAACGGAAUCCCAAAAGCAGCUG
MIMAT0000680	hsa-miR-106b-5p	MIMAT0000386	mmu-miR-106b-5p	0.55	UAAAGUGCUGACAGUGCAGAU
MIMAT0000752	hsa-miR-328-3p	MIMAT0000565	mmu-miR-328-3p	2.30	CUGGCCCUCUCUGCCCUUCCGU
MIMAT0000764	hsa-miR-339-5p	MIMAT0000584	mmu-miR-339-5p	2.17	UCCCUGUCCUCCAGGAGCUCACG
MIMAT0001340	hsa-miR-423-3p	MIMAT0003454	mmu-miR-423-3p	1.78	AGCUCGGUCUGAGGCCCCUCAGU
MIMAT0001631	hsa-miR-451a	MIMAT0001632	mmu-miR-451a	0.44	AAACCGUUACCAUUACUGAGUU
MIMAT0002177	hsa-miR-486-5p	MIMAT0014943	mmu-miR-486b-5p	0.47	UCCUGUACUGAGCUGCCCCGAG
MIMAT0004586	hsa-miR-15b-3p	MIMAT0004521	mmu-miR-15b-3p	0.49	CGAAUCAUUAUUUGCUGCUCUA
MIMAT0004672	hsa-miR-106b-3p	MIMAT0004582	mmu-miR-106b-3p	0.54	CCGCACUGUGGGUACUUGCUGC
MIMAT0004697	hsa-miR-151a-5p	MIMAT0004536	mmu-miR-151-5p	2.03	UCGAGGAGCUCACAGUCUAGU
MIMAT0004703	hsa-miR-335-3p	MIMAT0004704	mmu-miR-335-3p	2.45	UUUUUCAUUAUUGCUCCUGACC
MIMAT0000763	hsa-miR-338-3p	MIMAT0000582	mmu-miR-338-3p	2.01	UCCAGCAUCAGUGAUUUUGUUG
MIMAT0004762	hsa-miR-486-3p	MIMAT0017206	mmu-miR-486a-3p	0.51	CGGGGCAGCUCAGUACAGGAU
MIMAT0000736	hsa-miR-381-3p	MIMAT0000746	mmu-miR-381-3p	0.52	UAUACAAGGGCAAGCUCUCUGU
MIMAT0000432	hsa-miR-141-3p	MIMAT0000153	mmu-miR-141-3p	0.50	UAACACUGUCUGGUAAAGAUGG
MIMAT0000067	hsa-let-7f-5p	MIMAT0000525	mmu-let-7f-5p	0.65	UGAGGUAGUAGAUUGUAUAGUU
MIMAT0000757	hsa-miR-151a-3p	-	-	3.77	CUAGACUGAAGCUCCUUGAGG
MIMAT0004570	hsa-miR-223-5p	-	-	2.02	CGUGUAUUUGACAAGCUGAGUU
MIMAT0004600	hsa-miR-144-5p	-	-	0.47	GGAUAUCAUCAUAUACUGUAAG
MIMAT0004680	hsa-miR-130b-5p	-	-	1.97	ACUCUUUCCCUGUUGCACUAC
MIMAT0004951	hsa-miR-887-3p	-	-	2.40	GUGAACGGGCGCCAUCCCGAGG

MIMAT0022727	hsa-miR-1307-5p	-	-	3.06	UCGACCGGACCUCGACCGGCU
MIMAT0026478	hsa-miR-133a-5p	-	-	0.35	AGCUGGUAAAAUGGAACCAAAU
-	-	MIMAT0000161	mmu-miR-151-3p	1.97	CUAGACUGAGGCUCCUUGAGG
-	-	MIMAT0000569	mmu-miR-330-3p	2.42	GCAAAGCACAGGGCCUGCAGAGA
-	-	MIMAT0000609	mmu-miR-351-5p	1.78	UCCCUGAGGAGCCCUUUGAGCCU G
-	-	MIMAT0000904	mmu-miR-215-5p	2.28	AUGACCUAUGAUUUGACAGAC
-	-	MIMAT0003473	mmu-miR-133a-5p	0.33	GCUGGUAAAAUGGAACCAAAU
-	-	MIMAT0004186	mmu-miR-301b-3p	2.23	CAGUGCAAUGGUAUUGUCAAAG C
-	-	MIMAT0004532	mmu-miR-136-3p	0.54	AUCAUCGUCUCAAAUGAGUCUU
-	-	MIMAT0004583	mmu-miR-130b-5p	1.86	ACUCUUUCCCUGUUGCACUACU
-	-	MIMAT0004624	mmu-miR-15a-3p	0.41	CAGGCCAUACUGUGCUGCCUCA
-	-	MIMAT0004628	mmu-miR-21a-3p	1.65	CAACAGCAGUCGAUGGGCUGUC
-	-	MIMAT0004649	mmu-miR-339-3p	2.56	UGAGCGCCUCGGCGACAGAGCCG
-	-	MIMAT0004934	mmu-miR-872-5p	1.73	AAGGUUACUUGUUAGUUCAGG
-	-	MIMAT0004935	mmu-miR-872-3p	1.82	UGAACUAUUGCAGUAGCCUCCU
-	-	MIMAT0009441	mmu-miR-1968-5p	2.55	UGCAGCUGUUAAGGAUGGUGGA CU
-	-	MIMAT0014836	mmu-miR-3065-5p	1.85	UCAACAAAAUCACUGAUGCUGG
-	-	MIMAT0014851	mmu-miR-3071-3p	0.46	AUCAUCAAAACAAAUGGAGUCC
-	-	MIMAT0014944	mmu-miR-486b-3p	0.43	CGGGGCAGCUCAGUACAGGA
-	-	MIMAT0016988	mmu-miR-144-5p	0.45	GGAUAUCAUCAUAUACUGUAAG U
-	-	MIMAT0017018	mmu-miR-16-2-3p	0.45	ACCAAUAUUAUUGUGCUGCUUU
-	-	MIMAT0017056	mmu-miR-223-5p	1.97	CGUGUAUUUGACAAGCUGAGUU G
-	-	MIMAT0017169	mmu-miR-215-3p	2.02	UCUGUCAUUCUGUAGGCCAAU
-	-	MIMAT0020637	mmu-miR-5126	0.43	GCGGGCGGGGCCGGGGGCGGGG

Undepleted samples

human miRNA ID	human miRNA name	murine miRNA ID	murine miRNA name	FC	sequence
MIMAT0000068	hsa-miR-15a-5p	MIMAT0000526	mmu-miR-15a-5p	0.50	UAGCAGCACAUAAUGGUUUGUG
MIMAT0000069	hsa-miR-16-5p	MIMAT0000527	mmu-miR-16-5p	0.51	UAGCAGCACGUAAAUAUUGGCG
MIMAT0000081	hsa-miR-25-3p	MIMAT0000652	mmu-miR-25-3p	0.54	CAUUGCACUUGUCUCGGUCUGA
MIMAT0000087	hsa-miR-30a-5p	MIMAT0000128	mmu-miR-30a-5p	0.65	UGUAAACAUCCUCGACUGGAAG
MIMAT0000093	hsa-miR-93-5p	MIMAT0000540	mmu-miR-93-5p	0.57	CAAAGUGCUGUUCGUGCAGGUA G
MIMAT0000222	hsa-miR-192-5p	MIMAT0000517	mmu-miR-192-5p	1.67	CUGACCUAUGAAUUGACAGCC
MIMAT0000253	hsa-miR-10a-5p	MIMAT0000648	mmu-miR-10a-5p	1.68	UACCCUGUAGAUCCGAAUUUGU G
MIMAT0000424	hsa-miR-128-3p	MIMAT0000140	mmu-miR-128-3p	2.21	UCACAGUGAACCGGUCUCUUU
MIMAT0000425	hsa-miR-130a-3p	MIMAT0000141	mmu-miR-130a-3p	2.24	CAGUGCAAUGUUAAAAGGGCAU

MIMAT0000427	hsa-miR-133a-3p	MIMAT0000145	mmu-miR-133a-3p	0.40	UUUGGUCCCCUUCAACCAGCUG
MIMAT0000435	hsa-miR-143-3p	MIMAT0000247	mmu-miR-143-3p	0.63	UGAGAUGAAGCACUGUAGCUC
MIMAT0000436	hsa-miR-144-3p	MIMAT0000156	mmu-miR-144-3p	0.41	UACAGUAUAGAUGAUGUACU
MIMAT0000440	hsa-miR-191-5p	MIMAT0000221	mmu-miR-191-5p	2.49	CAACGGAAUCCCAAAAGCAGCUG
MIMAT0000752	hsa-miR-328-3p	MIMAT0000565	mmu-miR-328-3p	2.42	CUGGCCCUCUCUGCCCUUCCGU
MIMAT0001340	hsa-miR-423-3p	MIMAT0003454	mmu-miR-423-3p	2.21	AGCUCGGUCUGAGGCCCCUCAGU
MIMAT0001631	hsa-miR-451a	MIMAT0001632	mmu-miR-451a	0.44	AAACCGUUACCAUUACUGAGUU
MIMAT0002177	hsa-miR-486-5p	MIMAT0014943	mmu-miR-486b-5p	0.57	UCCUGUACUGAGCUGCCCCGAG
MIMAT0003218	hsa-miR-92b-3p	MIMAT0004899	mmu-miR-92b-3p	2.46	UAUUGCACUCGUCCCGGCCUCC
MIMAT0004597	hsa-miR-140-3p	MIMAT0000152	mmu-miR-140-3p	0.66	UACCACAGGGUAGAACCACGG
-	-	MIMAT0000161	mmu-miR-151-3p	2.05	CUAGACUGAGGCUCCUUGAGG
-	-	MIMAT0000609	mmu-miR-351-5p	2.35	UCCCUGAGGAGCCCUUUGAGCCUG
-	-	MIMAT0000904	mmu-miR-215-5p	2.27	AUGACCUAUGAUUUGACAGAC
-	-	MIMAT0014944	mmu-miR-486b-3p	0.63	CGGGGCAGCUCAGUACAGGA

supplementary table 4: miRNAs found differentially expressed in serum samples of mice carrying orthotopic xenografts versus tumor-free mice in depleted and undepleted samples (fold change cut-off = 1.5).

Supplementary table 5:

	3' adapter sequence	5' adapter sequence
**Illumina**	5' TGGAATTCTCGGGTGCCAAGG	5' GUUCAGAGUUCUACAGUCCGACGAUC
**NEBNext**	5' AAGATCGGAAGAGCACACGTCT	5' GUUCAGAGUUCUACAGUCCGACGAUC
**TailorMix**	5' TGGAATTCTCGGGTGCCAAGG	5' GTTCAGAGTTCTACAGTCCGACGATC

supplementary table 5: 3' and 5' adapter sequences of the TruSeq small RNA library preparation kit v2 (Illumina), the NEBNext Multiplex Small RNA library prep kit (New England Biolabs) and the TailorMix miRNA sample preparation kit v2 (Seqmatic) that were used for the preparation of the small RNA sequencing libraries.

Supplementary file 1: tRNA-halves depletion protocols

Beads-based protocol

1 hybridize probes and sample

1.1 Set heat block to 80 °C.
1.2 Add folowing components:

	high Input	low Input	
hybridization buffer (2x)	100	15	µl
tRNA probes (pmol/µl)	10	1	µl
RNase-free water		2	µl
RNA sample	variable	12	µl
		30	µl

each probe = 0.5 µM final concentration, make 2.5 µM worksolutions for the 5 probes

1.3 Mix by gentle vortexing and incubate at 80 °C for 2 min to denature RNA.
1.4 Transfer tube to cycler and allow to cool over period of 30 min to a temp of 22 °C, slow cooling promotes sequence-specific hybridization (program 0.1 °C/s, and keep 5 min)).
1.5 After cooling, briefly centrifuge the tube to collect sample and put on ice.

2 washing of beads

2.1 Resuspend beads in the vial (vortex for >30 sec or tilt and rotate for >5min) use the same volume of beads as sample in 1.5.
2.2 Transfer the desired amount of beads to a tube.
2.3 Add equal volume (or at least 1 ml) of washing buffer (=1X B&W buffer) and resuspend
2.4 place tube on magnet for 1 min, discard supernatant.
2.5 Remove tube from magnet and resuspend washed beads in the same volume of buffer as initiatial volume of beads.
2.6 Repeat steps 4-5 twice for a total of three washes, after third wash do not resuspend in B&W but continue to step 3.1.

3 prepare for RNA manipulation

3.1 Wash twice in solution A for 2 min, use same volume of solution A as het volume of beads or larger.
3.2 Wash once in solution B for 2 min, use same volume as with solution A.
3.3 Resuspend beads in 2x B&W buffer to a final 5 µg/µl concentration.

5 immobilization

5.1 Add sample to beads from step 4.2 or 3.3 (use the same volume of RNA sample as the volume of the beads).
5.2 Incubate at RT for 10 min with gentle mixing.
5.3 Briefly spin.
5.4 Place on magnet for 2 min.
5.5 **CRITICAL STEP:** Collect supernatant.

6 ethanol precipitation

6.1 Adjust the volume of each sample to 180 µl using RNase-free water.
6.2 Add 18 µl of 3 M sodium acetate to each tube.
6.3 Add 2 µl of glycogen (10 mg/ml) to each tube and mix by gentle vortexing.
6.4 Add 3 volumes (600 µl) of ice-cold 100 % ethanol to each tube and mix by gentle vortexing.
6.5 Place the tubes at -20 °C for at least 1 hour.
6.6 **CRITICAL STEP**: Centrifuge the tubes at > 10000 x g in a microcentrifuge for 30 minutes, carefully remove en discard the supernatant.
6.7 Wash the pellet with ice-cold 70 % ethanol and centrifuge at > 10000 x g for 5 minutes, carefully remove en discard the supernatant.
6.8 Repeat step 7 for total of two 70% ethanol washes.
6.9 **CRITICAL STEP**: Centrifuge briefly to collect any residual supernatant and carefully remove en discard the supernatant.
6.10 Allow pellet to air dry at room temperature for 5 minutes.
6.11 Dissolve the pellet in RNase-free water or buffer
6.12 Place on ice for immediate use, or keep at -20 °C overnight or -65 °C to -80 °C for longterm storage.

Beads buffers

**hybridization buffer**		
compound		
tris-HCl	0.1	M
NaCl	0.2	M

**solution A**		
compound		
DEPC-treated NaOH	0.1	M
		M
DEPC-treated NaCl	0.05	

2x B&W buffer		
compound		
tris-HCl	10	mM
EDTA	1	mM
NaCl	2	M

solution B		
compound		
DEPC-treated NaCl	0.1	M

Rnase H-based protocol

Hybridization

1. Prepare following mixture:

	low Input	
hybridization buffer (7.5x)	2	µl
tRNA probes (pmol/µl)	1	µl
RNA sample	12	µl
	15	µl

each probe = 0.5 µM final concentration, make 2.5 µM worksolutions for the 5 probes

2. Mix by pipetting up and down, spin down.
3. Place sample in thermal cycler:

temp	duration
80 °C	2 min
80-22 °C	0.1 °C/sec
22 °C	5 min hold

4. Spin down, place on ice.

RNase H digestion

5. On ice, prepare following master mix and mix by pipetting up and down:

component	volume	
RNase H	1	µl
RNase H reaction buffer (10x)	2	µl
DTT (0.1 mM)	2	µl

6. Add 5 µl of mix to the RNA sample from step 4
7. Mix by pipetting up and down
8. Place samples in thermocycler (lid 40 °C) and incubate at 37 °C for 30 min
9. Spin down and put on ice

DNase I digestion

10. On ice prepare follwing mix

DNase I reaction buffer	5	µl
DNase I	2.5	µl
nuclease-free water	22.5	µl

11. Add 30 µl mix to sample from step 9
12. Place in thermocycler (lid 40 °C) and incubate at 37 °C for 30 min
13. Spin down and place on ice

RNA purification by ethanol precipitation

14. Adjust the volume of each sample to 180 µl using RNase-free water.
15. Add 18 µl of 3 M sodium acetate to each tube.
16. Add 2 µl of glycogen (10 mg/ml) to each tube
    and mix by gentle vortexing.
17. Add 3 volumes (600 µl) of ice-cold 100 % ethanol to each tube
    and mix by gentle vortexing.
18. Place the tubes at -20 °C for at least 1 hour.
19. **CRITICAL STEP**: Centrifuge the tubes at > 10000 x g in a microcentrifuge for 30 minutes, carefully remove en discard the supernatant.
20. Wash the pellet with ice-cold 70 % ethanol and centrifuge at
    > 10000 x g for 5 minutes, carefully remove en discard the supernatant.
21. Repeat step 7 for total of two 70% ethanol washes.
22. **CRITICAL STEP**: Centrifuge briefly to collect any residual supernatant and carefully remove en discard the supernatant.
23. Allow pellet to air dry at room temperature for 5 minutes.
24. Dissolve the pellet in RNase-free water or buffer
25. Place on ice for immediate use, or keep at -20 °C overnight or -65 °C to -80 °C for longterm storage.

Rnase H buffers

RNase H reaction Buffer:			
**compound**	**1X**	**10X**	
KCl	0,075	0,75	M
tris-HCl	0,05	0,5	M
$MgCl_2$	0,003	0,03	M
DTT	0,01	0,1	M

pH 8.3 @ 25 °C

DNase I buffer			
**compound**	**1x**	**10x**	
tris-HCl	10	100	mM
$MgCl_2$	2,5	25	mM
$CaCl_2$	0,5	5	mM

pH 7.6 @ 25 °C

hybridization buffer 2		
**compound**		
tris-HCl	0,1	M
NaCl	0,2	M
